# Scalable Microscale Artificial Synapses of Lead Halide Perovskite with Femtojoule Energy Consumption

**DOI:** 10.1021/acsenergylett.4c02360

**Published:** 2024-11-08

**Authors:** Jeroen J. de Boer, Bruno Ehrler

**Affiliations:** Center for Nanophotonics, AMOLF, 1098 XG Amsterdam, The Netherlands

## Abstract

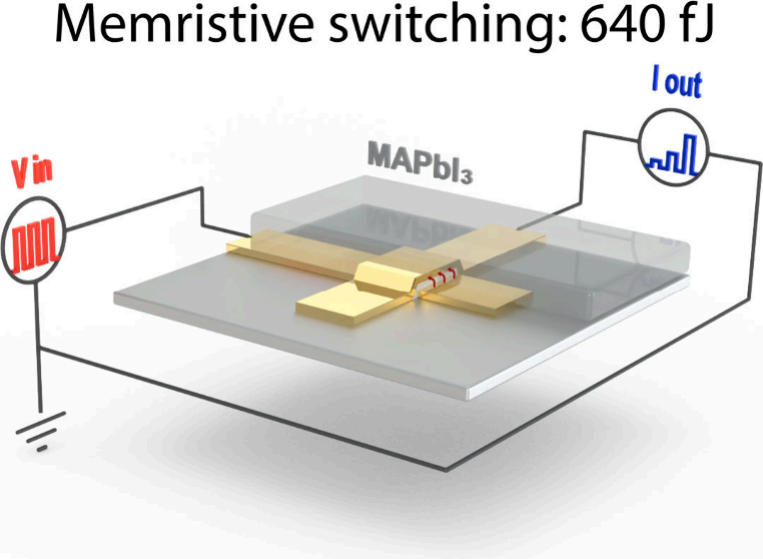

The efficient conduction of mobile ions in halide perovskites is highly promising for artificial synapses (or memristive devices), devices with a conductivity that can be varied by applying a bias voltage. Here we address the challenge of downscaling halide perovskite-based artificial synapses to achieve low energy consumption and allow high-density integration. We fabricate halide perovskite artificial synapses in a back-contacted architecture to achieve microscale devices despite the high solubility of halide perovskites in polar solvents that are commonly used in lithography. The energy consumption of a conductance change of the device is as low as 640 fJ, among the lowest reported for two-terminal halide perovskite artificial synapses so far. Moreover, the high resistance of the device up to hundreds of megaohms, low operating voltage of 100 mV and simple two-terminal architecture enable implementation in highly dense crossbar arrays. These arrays could potentially show orders of magnitude lower energy consumption for computation compared to conventional digital computers.

Recent years have seen the rapid development of ever more capable artificial intelligence (AI) models. These models now rival or even surpass human capabilities in a wide range of tasks, such as complex strategy games,^1−3^ image analysis,^4,5^ predicting protein folding^6^ or practicing law^7^ and medicine.^8^ While these feats are certainly impressive, the development comes with an exponential increase in computational demand and therefore power consumption.^9^ As an example, at the time of writing the generative pretrained transformer (GPT) models underlying ChatGPT are run on clusters ranging from eight up to thousands of GPUs each consuming up to 700 W of power.^10^ This large computational demand and power consumption is especially problematic for AI applications where relatively small devices, such as smart sensors, are required to function autonomously and without connecting to large external servers and power sources. By contrast, the most complex neural network we know, the human brain, only consumes roughly 20 W of power.^11^ One elegant solution to tackle this large discrepancy in power consumption is therefore to move to a novel way of computation that is inspired by the brain. In these so-called neuromorphic computing systems, electronic circuits are employed to mimic the functioning of biological neurons and synapses. Some well-known first implementations of this principle by Intel and IBM have demonstrated orders of magnitude reduction in power consumption for classification tasks already.^12,13^ The synapses in these neuromorphic systems were so far implemented by complementary metal-oxide semiconductor (CMOS) circuits.^14^ However, these circuits are bulky and typically take up most of the available area on the chip.^14,15^ Moreover, the energy consumption of tens to hundreds of pJ per synaptic event in the aforementioned neuromorphic chips^12,13^ is still significantly higher than the 1–10 fJ consumed by their biological counterpart.^16^

Memristive devices have recently gathered significant attention as an alternative building block of artificial synapses. These two-terminal devices have a resistance that can be varied by the application of a bias voltage and their working principle is typically based on formation of metallic filaments in metal oxides,^17,18^ a phase change from a nonconductive amorphous to a conductive crystalline material^19,20^ or polarization of a ferroelectric material.^21,22^ Their low energy consumption of operation down to the femtojoule range^17−22^ and the possibility to implement memristive devices in dense crossbar arrays^23,24^ make them an attractive alternative to synapses that are solely based on CMOS circuits. There are several requirements for memristive devices before they can effectively replace or be incorporated into CMOS-based artificial synapses. First, memristive devices with a range of switching speeds and state retention times are required to construct neuromorphic systems capable of learning and remembering of information.^25,26^ In addition, the resistance of the device should be high to prevent parasitic voltage drops on the interconnecting wires and to prevent electromigration of wire material.^23,24^ Lastly, large conductivity changes are required to help reduce read errors in downscaled devices with low operating currents.^24^

Recently, halide perovskites have been proposed as a novel material for implementation in memristive devices.^27−30^ Conductance changes in halide perovskite-based electronic devices are thought to originate from migration of ions or ion vacancies under the application of a bias voltage.^31^ The low activation energy of ion-migration in this class of materials means that their projected energy consumption is among the lowest of all memristive materials reported in literature, in the femtojoule range for device areas at or below 10 μm^2^.^28,32^ In addition, large changes in the conductance^29,33^ and the large range of time scales for conductance changes ranging from hundreds of milliseconds down to hundreds of picoseconds^28,33^ make halide perovskites attractive candidate materials for artificial synapses. However, so far few studies have focused on downscaling of halide perovskite memristive devices, which is a major challenge due to the high solubility of halide perovskites in polar solvents that are commonly used in lithography procedures.^34^ Downscaled halide perovskite devices with their promised femtojoule energy consumption have therefore not been demonstrated so far and there is currently no method to implement them in dense arrays on a chip. Moreover, downscaling of memristive devices based on other materials has previously been shown to result in higher operating voltages,^35−37^ leading to higher energy consumptions than expected based on the macroscale device. The lack of downscaled memristive devices of halide perovskites therefore makes it difficult to assess whether these materials retain their favorable resistance change properties for smaller device areas and hence to verify their scalability.^38^ In previous attempts, devices were downscaled by incorporation of halide perovskite in porous alumina membranes or in holes in a SiO_2_ layer with top contacts evaporated through a shadow mask. However, with these approaches, the energy consumption of conductance changes was still on the order of several picojoules and the device geometry is difficult to scale to large networks.^33,39^

Here we report a method to downscale halide perovskite artificial synapses to the microscale to reach an energy consumption of conductance changes down to 640 fJ. The synapse is operated at low voltages of 100 to 200 mV with large conductance changes up to 5 orders of magnitude. Moreover, the synapse has a switching speed on the order of tens of milliseconds and a retention time of tens of seconds, similar to biological synapses. The time scales of conductance changes differ significantly from those of synapses based on metal oxides, phase change materials and ferroelectrics and therefore complements these existing memristive devices. The high resistance up to hundreds of megaohms and the two-terminal architecture make our devices ideal for integration in high density crossbar arrays.

The back-contacted, two-terminal device architecture that was adopted for the downscaled synapses is shown schematically in Figure 1. The device consists of two gold electrodes that form a crosspoint with a SiO_2_ spacer that separates the electrodes. Methylammonium lead iodide (MAPbI_3_) perovskite is spin coated over the electrodes and forms the active layer of the device. By depositing both electrodes prior to perovskite deposition, our device avoids processing on top of the relatively sensitive perovskite layer. Depending on the bias voltage applied to the electrodes, current flows through the MAPbI_3_ layer from the top of the bottom electrode to the sides of the top electrode or vice versa. Bias voltages applied to the MAPbI_3_ layer induce hysteresis that modulates the resistance of the device, mimicking the plasticity of biological synapses.^40^ Embedded in a network, one of the electrodes would electronically connect to the presynaptic neuron that sends voltage pulses to the postsynaptic neuron via the other electrode of this synapse.

**Figure 1:**
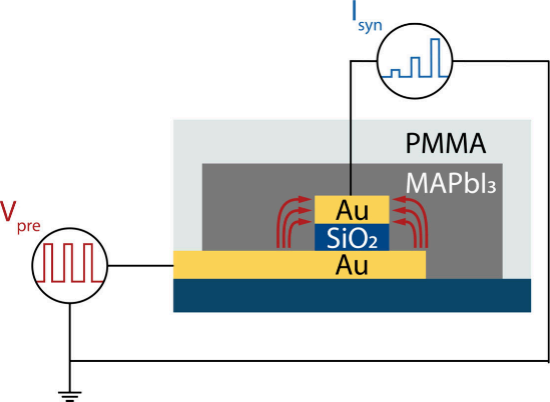
Schematic representation of the artificial synapse. The device consists of two gold electrodes that form a crosspoint and sandwich a SiO_2_ spacer layer. A MAPbI_3_ active layer is spin-coated over the electrodes. Bias-voltage-induced hysteresis leads to a change in the postsynaptic current I_syn_ of the device with each successive presynaptic voltage pulse V_pre_ that is applied.

The device fabrication procedure is outlined in Figure 2 a-e. First, the gold bottom electrodes were patterned on a Si/SiO_2_ (100 nm) substrate by a UV-lithography procedure. A 60 nm SiO_2_ layer was then deposited on the bottom electrode by inductively coupled plasma chemical vapor deposition (ICPCVD). Top electrodes were subsequently patterned on the SiO_2_ layer by a second UV-lithography step, aligned perpendicular to the bottom electrode. The SiO_2_ layer acts as an insulating layer that prevents a short-circuit between the top and bottom electrodes. The top electrodes were now used as a hard mask for reactive ion etching (RIE) of the SiO_2_ layer. An optical microscopy and scanning electron microscopy (SEM) image of the crosspoint of the electrodes after the RIE of the SiO_2_ layer are shown in Figure 2f. Finally, the MAPbI_3_ active layer and a PMMA capping layer were spin-coated onto the substrate. An X-ray diffraction (XRD) pattern and SEM image of a spin-coated MAPbI_3_ film are given in Figure S1 of the Supporting Information. Spin coating the halide perovskite layer only in the final step prevents degradation of the perovskite layer due to exposure to polar solvents used in the lithography procedure. Moreover, the encapsulation with PMMA has been shown to significantly reduce the rate of degradation of the perovskite layer under ambient conditions and at elevated temperatures.^41^

**Figure 2:**
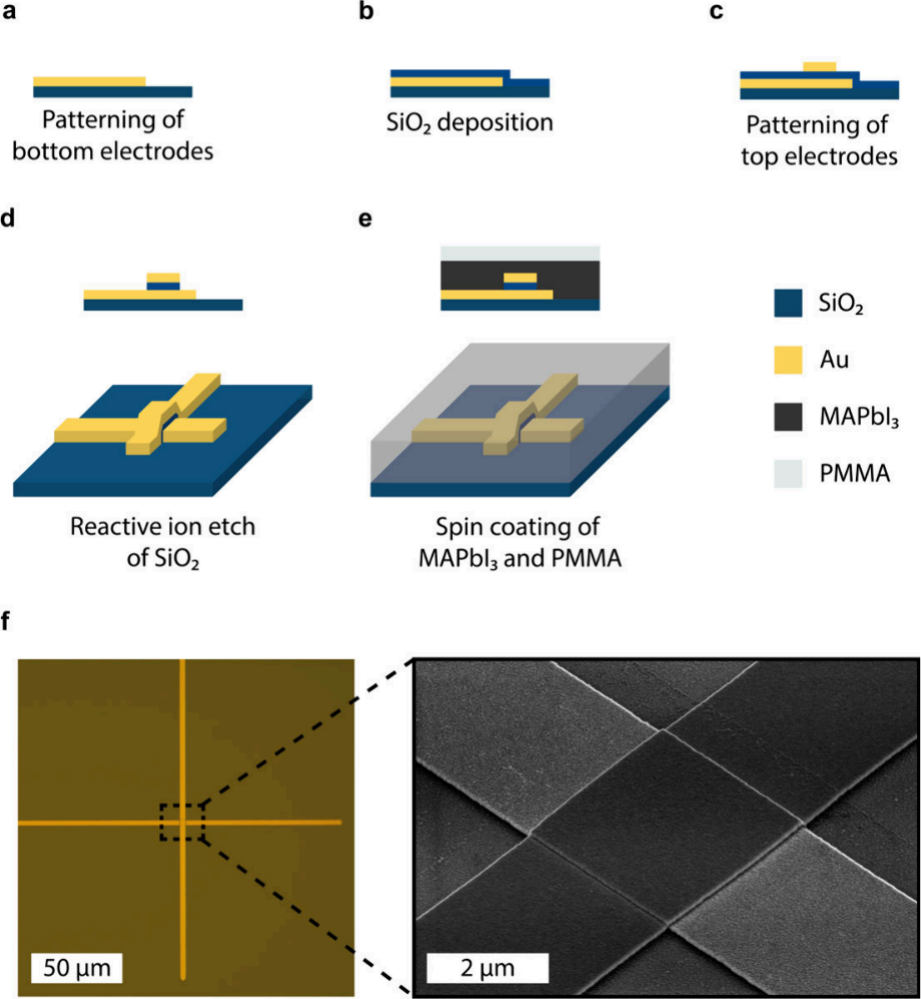
Fabrication procedure of the perovskite synapses. (a) 2.5 μm wide Au bottom electrodes are patterned on a thermally oxidized Si substrate by UV lithography. (b) A 60 nm insulating SiO_2_ spacer is deposited by ICPCVD. (c) 2.5 μm Au top electrodes are patterned perpendicular to the bottom electrode in a second UV lithography step. (d) The top electrode is used as a hardmask during removal of SiO_2_ from the bottom electrode with a reactive ion etch. (e) The perovskite active layer and a PMMA capping layer are spin-coated in the final fabrication step. (f) Optical microscopy and tilted SEM image of the crosspoint of two 2.5 μm electrodes.

An I–V curve of the device before perovskite deposition was measured to ensure that the SiO_2_ spacer does not form a shunt in the final device with the perovskite layer. As can be observed in Figure S2, the current that flows between the electrodes through the SiO_2_ spacer falls below the detection limit of our measurement setup for all bias voltages used in this work. In addition, no resistance changes were measured up to 3 V without the perovskite. We can therefore exclude contributions of the SiO_2_ layer to resistance changes of the final device.

The geometry of the current flow through the MAPbI_3_ layer from the top of the bottom electrode to the sides of the top electrode makes the exact device area difficult to define. However, device volume can still be minimized by decreasing the width of the electrodes. All devices discussed in the rest of this work contained gold top and bottom electrodes that were 2.5 μm wide. This electrode width was chosen as a compromise allowing for high fabrication yields and minimized device area. A clear advantage of our crossbar geometry is that the dimensions of the halide perovskite film do not limit the device dimensions and therefore the crosspoints can be implemented in dense arrays underneath a single, macroscopic film.

Thirty-five I–V curves demonstrating the typical conductance change behavior of the microscale device are shown in Figure 3a. The current rapidly increases by approximately three to 5 orders of magnitude when a potential of 0.1 to 0.2 V is reached in the forward sweep from 0 to 0.2 V. The device remains in this higher conductive state with conductance S_ON_ in the reverse sweep from 0.2 to −0.2 V and is reset to the lower conductive state with conductance S_OFF_ between 0 and −0.2 V. Similar rapid conductance changes of several orders of magnitude have been reported before in macroscopic perovskite memristive devices. Interestingly, for macroscale devices these changes typically occur at higher voltages than those reported here and are attributed to the formation of conductive filaments through the film.^42−44^ One of the I–V sweeps is shown in Figure S3, plotted on the linear scale. From this measurement it follows that the synapse shows Ohmic conduction after the conductance increase, which is expected after the formation of a conductive filament through the bulk of the film.^45^ The I–V sweep therefore suggests that the measured conductance changes in our device are due to the formation and rupture of a conductive filament as well. Formation of these conductive filaments in metal-halide perovskite-metal devices is well-established and has been demonstrated experimentally in previous reports.^43,44,46,47^ The lower voltages at which the conductance changes are observed can be explained by the shorter distance between the electrodes than those typically used in macroscale devices,^42−44^ resulting in proportionally larger electric field strength. In the I–V sweep in Figure S3, the Ohmic response of the device is not maintained for negative voltages, which we ascribe to the relative instability of the filament and the large electric field experienced by the filament, even at low applied voltages. Importantly, these measurements show that halide perovskite synapses maintain low operating voltages after downscaling. We note that conductance changes can also occur for negative applied voltages, as demonstrated in Figure S4. The device is symmetric, but switches preferentially in the direction of the initial voltage sweep. This behavior is consistent with the conductive filament mechanism.^45^ Once the filament is formed in one direction, the field within the device is small. Only after the rupture of the filament can the voltage drop in the bulk of the device be large enough to grow a new filament in the reverse direction. The preference of switching in one sweeping direction that we show here is consistent with previous work on symmetric metal-halide perovskite-metal devices.^44^

**Figure 3:**
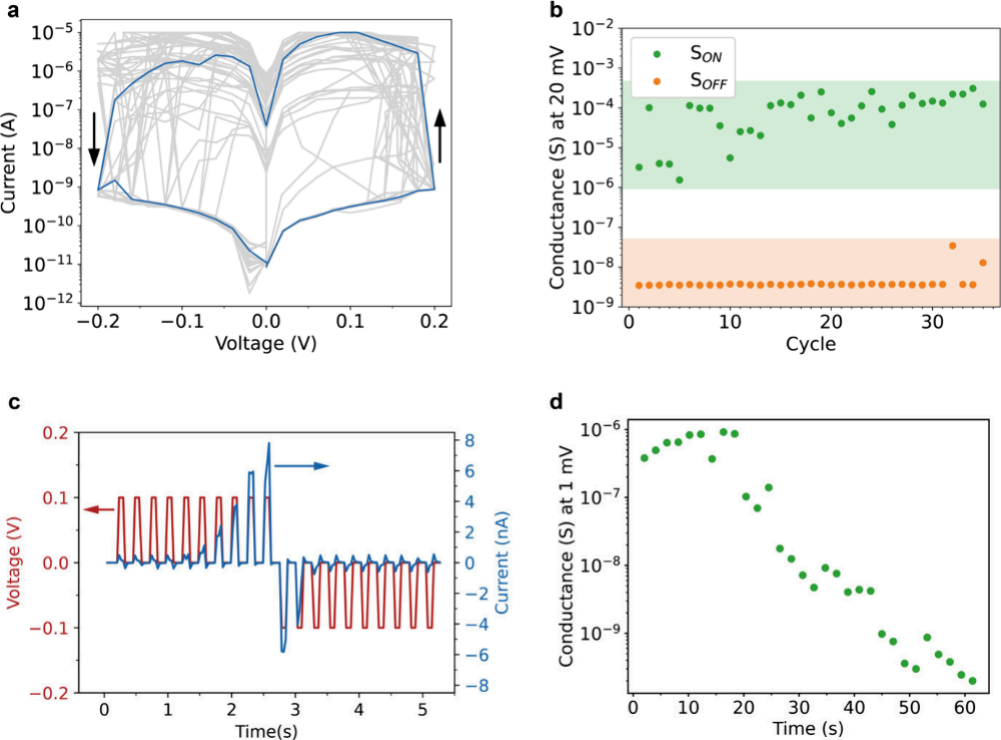
Conductance changes of the synapse. (a) The median (blue) of 35 I–V sweeps (gray) of the synapse, showing a rapid increase of the current between 100 and 200 mV. (b) The conductance in the forward (S_OFF_) and backward (S_ON_) sweeps, calculated from the current measured at 0.02 V in the I–V sweeps in (a). An increase in the conductance of 3 to 5 orders of magnitude is observed for each cycle. (c) Pulsed I–V measurements demonstrating the reversible conductance changes with ten 80 ms pulses of +100 mV, followed by ten pulses of −100 mV and of the same duration. (d) Retention time measured directly after switching the conductance of the synapse to S_ON_ with 10 pulses (200 mV, 80 ms). The retention time is measured with a 1 mV probe pulse. The conductive state is stable for tens of seconds.

The conductance values at 0.02 V for the forward and the backward sweeps were calculated by dividing the measured current by this voltage (Figure 3b). In each of the cycles the device shows a large S_ON_ to S_OFF_ ratio between 3 and 5 orders of magnitude and a low conductance down to the nS range, which is important for the scalability of the device.^23,24^ Although the conductance in the S_OFF_ state is consistent between cycles, there is some variation in the conductance in the S_ON_ state. There is no clear trend of decreasing or increasing S_ON_ with each successive cycle. Therefore, the variation is unlikely due to degradation of the device, but probably due to a stochastic nature of the resistance changes.^26^

The distribution of the voltages where conductance changes, the S_OFF_ and S_ON_ state conductance, and the S_ON_ to S_OFF_ ratio are given in Figure S5 of the Supporting Information. The voltages at which the conductance is switched on and off are 0.16 ± 0.03 V and −0.12 ± 0.06 V, respectively. Despite these low switching voltages, the device shows a remarkably high S_ON_ to S_OFF_ ratio of 2.7 ± 2.2 × 10^4^.

To investigate the switching behavior, we applied a pulsed voltage. Voltage pulses produced more gradual conductance changes, as demonstrated in Figure 3c. Ten consecutive voltage pulses of 0.1 V and 80 ms in duration were applied to the device, followed by ten consecutive pulses with the same duration, but of opposite polarity. The measured current during the application of the positive voltage pulses increased from 0.1 nA in the first pulse to 8 nA in the tenth pulse. During the subsequently applied negative voltage pulses, the current changed from −6 nA in the first negative pulse to −0.1 nA in the tenth pulse, indicating a decrease in the conductance of the device. The pulsed measurement demonstrates the change of the conductance of the device over orders of magnitude upon application of a bias voltage. In addition, the measurement shows that several conductive states are accessible between the S_ON_ and S_OFF_ states demonstrated in Figure 3b. This tunability of the conductive state of the synapse is analogous to the tunability of the connection strength of biological synapses, where several states are accessible depending on the degree of potentiation of the synapse.^48^

The retention time of the S_ON_ state was determined by applying periodic 1 mV probe pulses immediately after setting the device in the S_ON_ state. This voltage is too low to cause conductance changes of the device, as can be observed in Figure S6a. The evolution of the conductance over time is shown in Figure 3d, while the full measurement is given in Figure S6b. The conductive state does not decrease for the first 20 s of the measurement, after which the conductance starts to decay to the S_OFF_ state, which is reached 30 s after the start of the measurement. Similar time constants for changing and retention of the state of the synapse have been reported for biological synapses.^40^

I–V curves and the corresponding S_ON_ and S_OFF_ values at 20 mV of different devices are shown in Figure S7. From these IV curves it follows that devices from different batches all show similarly large conductance changes of several orders of magnitude with an onset between 200 and 400 mV, demonstrating the reproducibility of our fabrication procedure.

One of the promises of perovskite artificial synapses is that their energy consumption might be very low, approaching biological synapses. We reduced the voltage pulse duration to 55 ms to reduce the dissipated energy during a conductance change of the synapse. At such short time, and at the low current measured, the parasitic capacitance of our measurement setup introduced a significant measurement artifact, as is evident when comparing the measured data of the synapse in Figure S8a and Figure S8b with data measured without contacting the sample in Figure S8c. We therefore corrected for this parasitic displacement current by subtracting the current measured without contacting the synapse from the measured data. The corrected pulsed measurement is shown in Figure 4a. The mean current determined at each of the ten pulses is plotted in Figure 4b. The current increased from 0.1 nA in the first pulse to 10 nA in the final pulse, similar to the currents measured for the longer pulses (Figure 3c). Both the exponential increase of the current with each successive pulse,^45^ and the fact that not each pulse brings about the same relative increase in the output current are expected for a memristive device where the inherently stochastic growth of a filament causes changes in the conductance.^49^ Moreover, the figure again highlights that several conductive states can be accessed between the S_OFF_ and S_ON_ state by applying consecutive voltage pulses to the device. Although artificial synapses based on other materials have shown a larger number of accessible states,^20^ our device still demonstrates the analog conductance changes that are reminiscent of biological synapses.^40,48^

**Figure 4:**
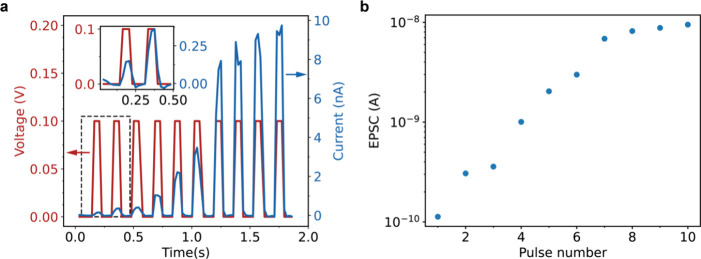
Performance characteristics of the artificial synapse. (a) Pulsed I–V measurements with 0.1 V, 55 ms pulses. An increase in the conductance of 2 orders of magnitude is measured between the first and tenth pulses. The inset highlights the first two pulses of the measurement in the dotted rectangle where approximately 640 fJ of energy is consumed to double the output current. (b) Average excitatory postsynaptic current (EPSC) at each of the spikes in (a)..

The inset of Figure 4a and the first two data points in Figure 4b show that the conductance of the synapse is approximately doubled in the second pulse. To calculate the energy consumption *E* of this doubling of the conductance change we take the product of the measured current *I*, applied voltage *V* and pulse duration *t*, *E* = *IVt*, yielding an energy consumption of 640 fJ. Considering the large 2-fold increase in the conductance, we expect that a further decrease of the energy consumption is possible by decreasing the pulse duration or magnitude of the voltage. The energy consumption is approaching values measured for biological synapses, which is especially promising in our scalable device architecture that could allow for the fabrication of entire microscopic artificial neural networks on a chip.

Femtojoule energy consumptions have been reported in previous work on macroscale halide perovskite artificial synapses,^43,50,51^ but in those cases the energy consumption of the read pulses was considered and not of the conductance change itself, as we do here. In the final network the energy consumption of the conductance update will be a significantly larger contributor to the total energy consumption of the synapse compared to the read pulse.^12^ In addition, the energy consumption of a read pulse can be made arbitrarily small by applying a pulse with the shortest possible time and voltage amplitude. We therefore think it is more appropriate to consider the energy consumption of the conductance update when assessing the energy consumption of the synapse. We are aware of only one work where a lower energy consumption, of tens of femtojoules, was reported for a conductance change of a halide perovskite synapse.^52^ However, in this work devices were fabricated with a lateral architecture, which is not suitable to achieve high device densities on the final chip.^38^ Moreover, the distance between the electrodes in this work was 100 μm. Downscaling of these devices for high device densities on a chip will require a smaller distance between the electrodes, which will likely increase the current and therefore energy consumption of the devices significantly.^53^

Energy consumptions of conductance changes in the femtojoule range have been reported for memristive devices based on filament growth in metal oxides,^17^ phase change materials^19^ and ferroelectrics^22^ as well. However, device areas were significantly smaller in these earlier reports already. Assuming a linear decrease of the energy consumption with decreasing device area, we estimate that for similar device areas we can reach orders of magnitude lower energy consumptions with our device architecture, as illustrated by Figure 5a. Only three-terminal transistor versions of artificial synapses based on doping of an organic semiconductor have been reported to reach significantly lower energy consumptions for a given device area.^53,54^ Nevertheless, for these devices typically only the drain-source current is considered when calculating the energy consumption of the synapse, while the gate-source current due to leakage currents and capacitive charging is ignored. Taking into account this extra contribution to the energy consumption of the device would likely give significantly larger energy consumptions of these synapses. Apart from that, the three-terminal architecture is less scalable due to the incompatibility with high density crossbar arrays, unlike the simpler two-terminal architecture of the synapse presented here.^23,24^ Moreover, the organic artificial synapses only achieved S_ON_ to S_OFF_ ratios of up to 1 order of magnitude,^53,54^ while the synapse in our work reaches S_ON_ to S_OFF_ ratios of 3 to 5 orders of magnitude. In fact, the S_ON_ to S_OFF_ ratio we report here is among the highest of those reported for energy-efficient artificial synapses, as can be seen in Figure 5b. This high S_ON_ to S_OFF_ ratio is important for the accuracy of computation, in particular for further downscaled devices with lower operating currents.^24^

**Figure 5:**
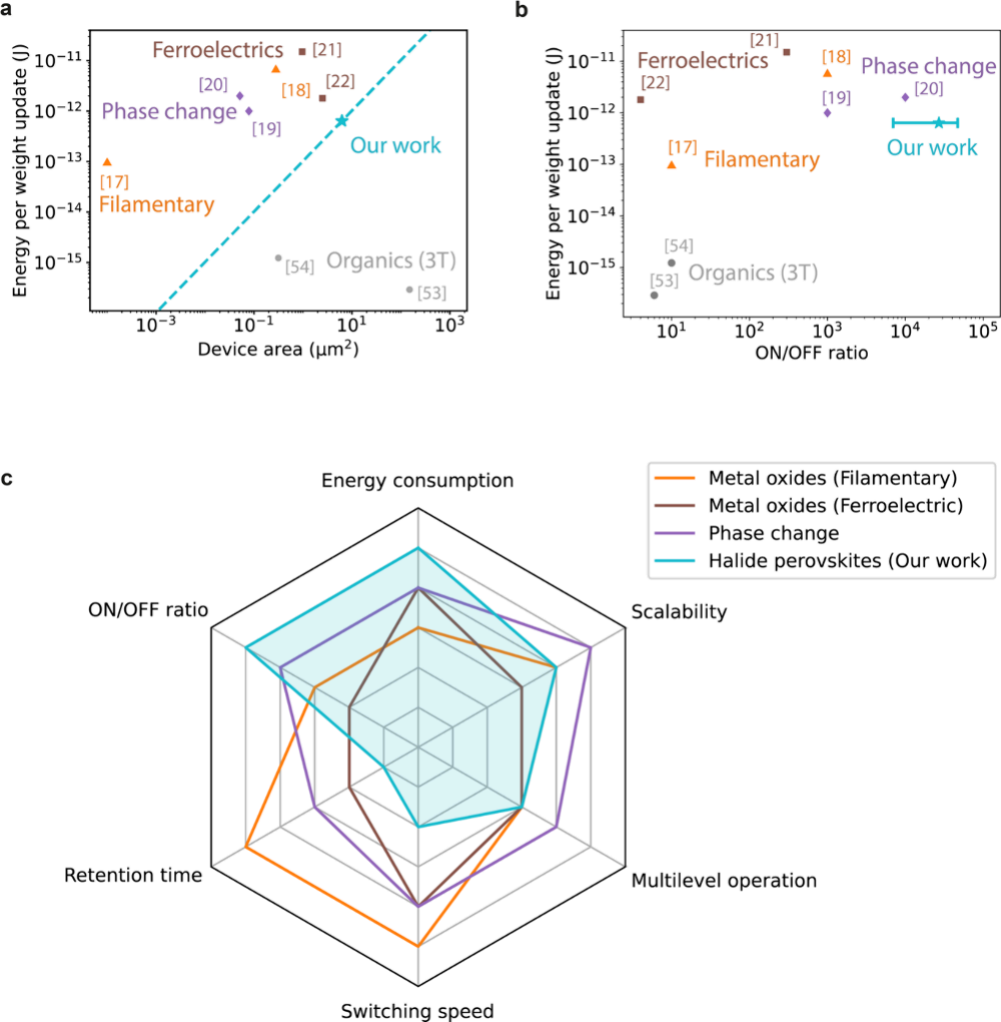
Comparison of the halide perovskite-based artificial synapse with low-energy consumption artificial synapses based on other materials. Energy efficiency with respect to (a) the device area and (b) ON/OFF ratio of the synapse compared to values reported in previous work. The error bar represents one standard deviation. (c) Comparison of the halide perovskite-based artificial synapse with two-terminal low-energy consumption artificial synapses based on other materials in terms of several key characteristics of artificial synapses. Supplementary Note 3 explains how the figure was compiled.

It should be noted that crosstalk between devices could occur with our current device layout if devices are implemented in high density. However, we show in Supplementary Note 2 that lateral devices with a 90 nm distance between the electrodes do not show the same changes in the resistance over orders of magnitude. Hence, crosstalk should not be an issue for lateral distances of at least 90 nm between devices.

Figure 5c compares the performance characteristics of the artificial synapse presented in this work with two-terminal artificial synapses based on the other materials presented in Figure 5a and Figure 5b. Compared to the previously reported synapses, the artificial synapse presented in this work excels in terms of energy consumption and its simultaneously high ON/OFF ratio. The halide perovskite synapse has a switching speed on the order of tens of milliseconds and a retention time of tens of seconds. As efficient processing of data by neuromorphic hardware requires synapses with switching speeds and state retention times that are well-matched to those of the incoming data,^14,55^ these synapses are well suited for processing input signals such as speech or gestures that are received at a low rate.^56^ Moreover, memristive elements with a large range of time constants for switching and state retention are required to design neuromorphic circuits that efficiently emulate the different forms of plasticity in the brain and to enable both learning and remembering of information by the same network.^12,25,26,56^ The synapse we present here therefore nicely complements metal oxide resistive switching,^17,18^ phase change^19,20^ and ferroelectric^21,22^ synapses for which faster switching speeds and longer retention times were reported.

In conclusion, we have described an artificial synapse with an energy consumption as low as 640 fJ, high ON/OFF ratio, with time constants for switching and state retention that are similar to those of biological synapses and that are complementary to existing downscaled artificial synapses based on other materials. Additionally, the synapse retained the low switching voltages and large conductance changes when scaled down, which proves that halide perovskite based artificial synapses can be scaled effectively at least to the microscale. This device is enabled by a UV-lithography procedure to fabricate back-contacted halide perovskite artificial synapses on the microscale. The back-contacted architecture allows deposition of the halide perovskite material in the final step and thereby prevents degradation of the perovskite layer. Further downscaling of the device might reduce the energy consumption even further, potentially making the halide perovskite synapse the most energy efficient of all existing two-terminal devices. Moreover, the large conductance changes up to 5 orders of magnitude, large resistance up to hundreds of megaohms combined with the low 100 mV operating voltage and simple two-terminal architecture make the synapse promising for integration in dense crossbar arrays.

## Supporting Information Available

Fabrication procedure of the back-contacted artificial synapse, MAPbI_3_ film characterization, I–V sweep of the device without a MAPbI_3_ active layer, highlighted single I–V sweep of the synapse, retention time measurement, I–V sweeps of different devices demonstrating reproducibility, pulsed I–V data with parasitic capacitance, note on crosstalk for high integration densities (PDF)
