# Supplementary material for: Scalable Microscale Artificial Synapses of Lead Halide Perovskite with Femtojoule Energy Consumption
Source: ACS Energy Lett. 2024 Nov 8;9(12):5787–94. doi: 10.1021/acsenergylett.4c02360 (PMC11650764; doi:10.1021/acsenergylett.4c02360)
Supplement: Supplementary file 1 — nz4c02360_si_001.pdf [file nz4c02360_si_001.pdf]

Supplementary Information to

Scalable microscale artificial synapses of lead halide perovskite with femtojoule energy consumption

Jeroen J. de Boer[1], Bruno Ehrler[1,*]
[1]*Center for Nanophotonics, AMOLF, 1098 XG, Amsterdam, the Netherlands*

Supplementary Note 1. Fabrication procedure of the back-contacted artificial synapse

Materials

Si wafers were purchased from Siegert Wafer. PbI_2 (99.99%) was purchased from TCI. Methylammonium iodide (MAI) was purchased from Solaronix. Anhydrous DMF and chlorobenzene were purchased from Sigma-Aldrich. 950 PMMA A8 was purchased from Kayaku Advanced Materials. All materials were used without further purification.

Fabrication of the back-contacted artificial synapse

Devices were fabricated on Si wafers with a 100 nm thermal oxide layer. Gold bottom electrodes were patterned on the wafer with a lift-off procedure with MA-N1410 photoresist. UV exposure with a Süss MA6/BA6 mask aligner was followed by development in MA-D533/s. A 5 nm Cr adhesion layer and an 80 nm Au electrode layer were deposited on the patterned resist by e-beam physical vapor deposition. Lift-off was then performed by soaking in acetone for one hour. A 60 nm SiO_2 was deposited from a O_2 and SiH_4 gas mixture using ICPCVD in an Oxford PlasmaPro100 ICPCVD system. Gold top contacts were patterned using the same procedure as for the bottom electrodes. After patterning of the top electrodes, the SiO_2 layer was etched in an Oxford Plasmalab 80 Plus system with an Ar and CHF_3 gas mixture, using the top electrodes as a hardmask.

Inside a nitrogen filled glovebox (< 0.5 ppm O_2 and water), a stoichiometric mixture of PbI_2 and MAI was dissolved in DMF to obtain a 40 wt% $MAPbI_3$ precursor. The precursor was spin coated over the electrodes at 4000 rpm for 30 seconds in the same glovebox. Chlorobenzene was added as an antisolvent after 3 seconds of spinning. Directly after spin coating the samples were annealed at 100 °C for 10 minutes. The 950 PMMA A8 solution was spin coated on top of the halide perovskite at 3000 rpm for 45 seconds, followed by a 5 minute bake at 100 °C.

Fabrication of lateral devices

Lateral devices were fabricated following the same procedure as for the back-contacted devices, but using an e-beam lithography procedure to pattern an ARP 6200 resist layer. A Raith Voyager lithography system was used to pattern the electrodes, wires and contact pads. The

same subsequent metallization, lift-off and halide perovskite spin coating procedures were followed as for the back-contacted devices.

$MAPbI_3$ film characterization

X-ray diffraction measurements were performed on a Brucker D2 PHASER with a 1.54184 Å Cu K_α source using a 2θ = 0.025° step size and a 0.100 second exposure time per step. SEM images were taken on a FEI Verios 460 with a 5.00 kV acceleration voltage and a 100 pA beam current.

Electrical characterization

I-V curves between -0.2 and 0.2 V were measured with a Keithley 4200A-SCS Parameter Analyzer. Voltage pulses were applied with a Keysight B2902A Precision Source/Measure Unit. Pulses of 100 mV and a 80 to 55 ms pulse width were applied to change the conductivity of the device. For retention time measurements, 600 ms 1 mV pulses were applied after setting the conductivity of the device to read out the conductive state.

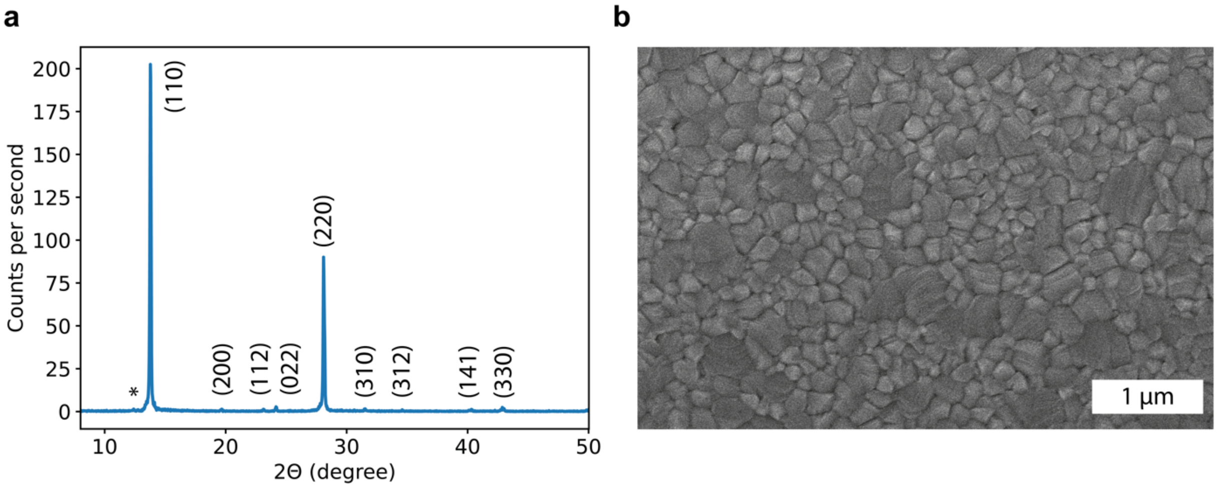

Figure S1. Characterization of the spin-coated $MAPbI_3$ film. ***(a)*** *XRD pattern of the film. Each peak is annotated with the assigned crystal plane of the $MAPbI_3$ lattice. We attribute the peak indicated with the asterisk to the (001) plane of PbI_2.* ***(b)*** *SEM image of the film, indicating that the crystallites in the film are between 100 and 500 nm in diameter.*

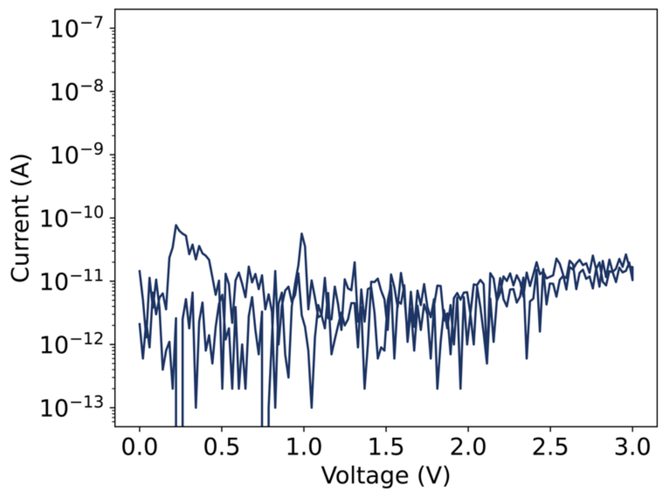

Figure S2. IV-sweep between 0 and 3 V of the device without a perovskite layer. The current remains at the resolution limit of the measurement setup over the whole voltage range.

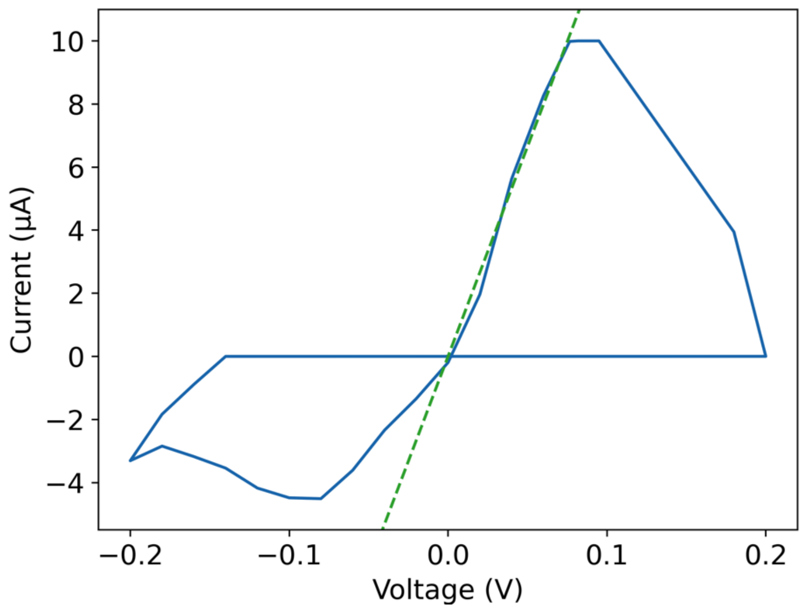

Figure S3. The I-V sweep of cycle 8 in Figure 3a, plotted on a linear scale. The region from the backwards sweep after the conductance change was fitted with Ohm's law. The obtained fit corresponded to a conductance of 133 µS and is shown as the dashed green line. The fact that the region is described well by Ohm's law (R^2 = 0.99) suggests that the conductance change is caused by the formation of a conductive filament in the perovskite film.

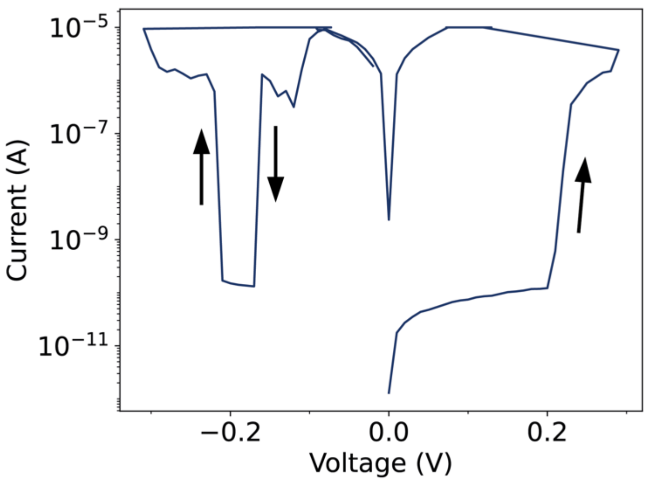

Figure S4. I-V sweep of the device with higher voltage amplitudes compared to Figure 3 of the main text. After setting and resetting the device at voltages similar to those in Figure 3, the conductance of the device increases rapidly for the negative sweeping direction as the voltage is further increased in the negative direction.

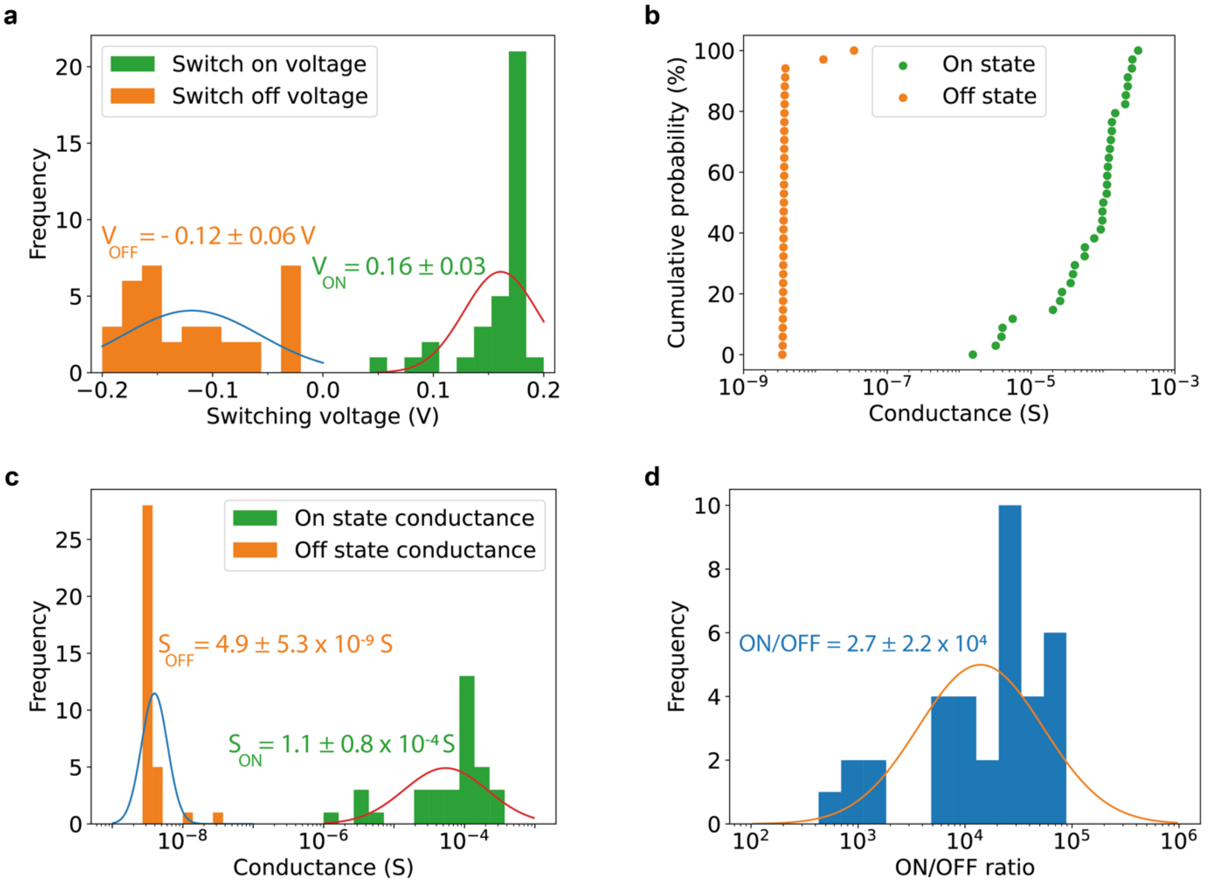

Figure S5. Statistical analysis of the conductance changes of the synapse, based on the 35 I-V curves in Figure 3a in the main text. ***(a)*** *Histogram of the voltages at which the current switches to over (V_{ON}) or below (V_{OFF}) 10^{-8} A, with the corresponding fit to a normal distribution, mean and standard deviation. The cumulative probability* ***(b)*** *and the histogram, with fits to a log-normal distribution, the mean and standard deviation* ***(c)*** *of the different S_{OFF} and S_{ON} states.* ***(d)*** *A histogram of the S_{ON}/S_{OFF} ratio, with a fit to a log-normal distribution, the mean and standard deviation.*

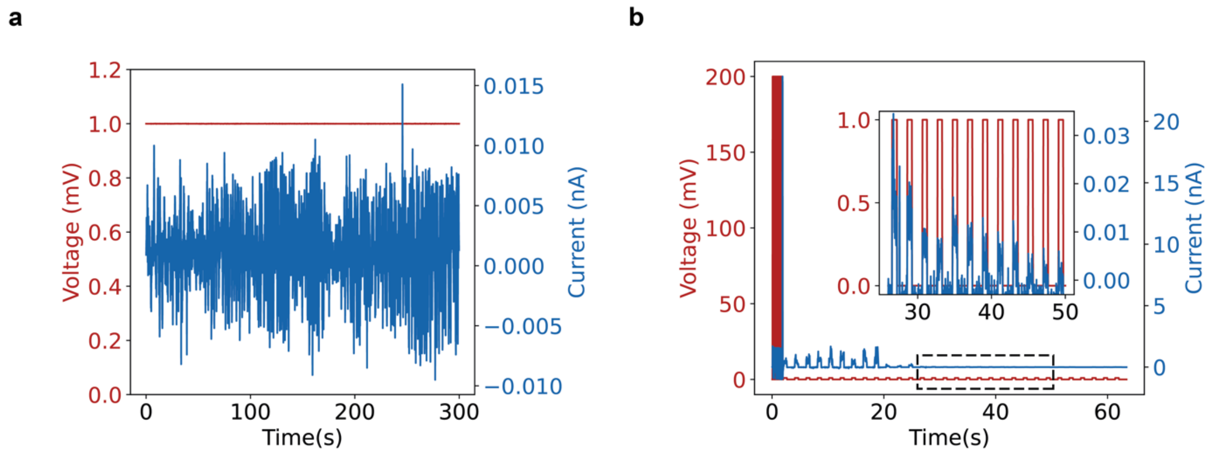

Figure S6. Retention time measurements of the synapse. ***(a)*** *A constant potential of 1 mV is applied to the synapse for 300 seconds, which does not lead to any measurable change in the conductance.* ***(b)*** *Pulsed measurement where the conductance of the halide perovskite artificial synapse is tracked by applying periodic 1 mV pulses to the device, after an initial 200 mV pulse train to set the device in the S_{on} state. The inset shows the pulsed measurement in the dotted rectangle after the initial 25 seconds.*

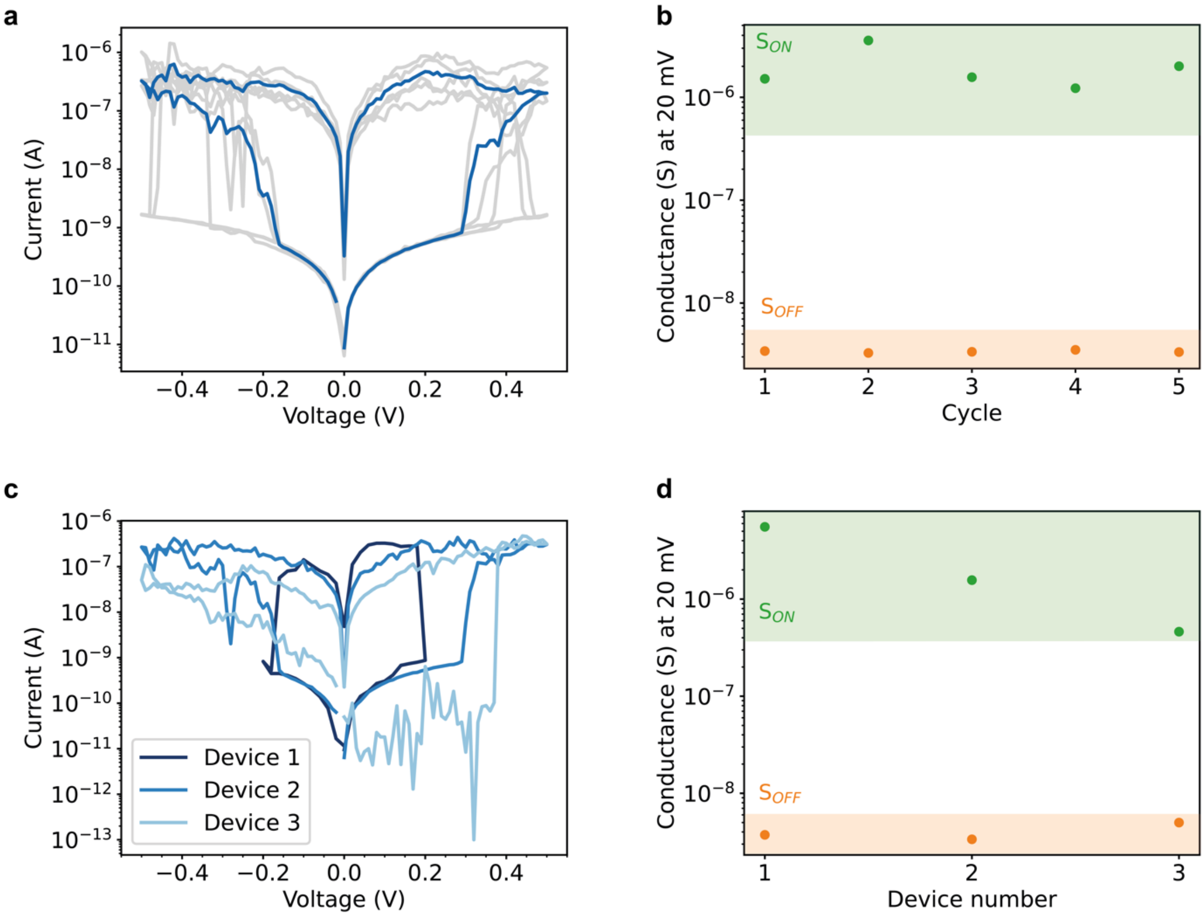

Figure S7. IV-curves of artificial synapses from different batches. ***(a)*** *The average (blue) of five IV-curves (grey) measured from a device different to the one in the main text. The IV-curves demonstrate a similar rapid increase in the current at approximately 300 - 400 mV as shown in Figure 3a.* ***(b)*** *The conductance in the forward (S_{OFF}) and backward (S_{ON}) sweep, calculated from the current measured at 0.02 V in the I-V sweeps in* ***(a)****.* ***(c)*** *I-V curves of three devices from different batches and* ***(d)*** *the conductance in the forward (S_{OFF}) and backward (S_{ON}) sweep, calculated from the current measured at 0.02 V for each of the three devices. All curves show a similar rapid increase in the current, with an onset between approximately 200 and 350 mV, with a large conductance change of two to three orders of magnitude.*

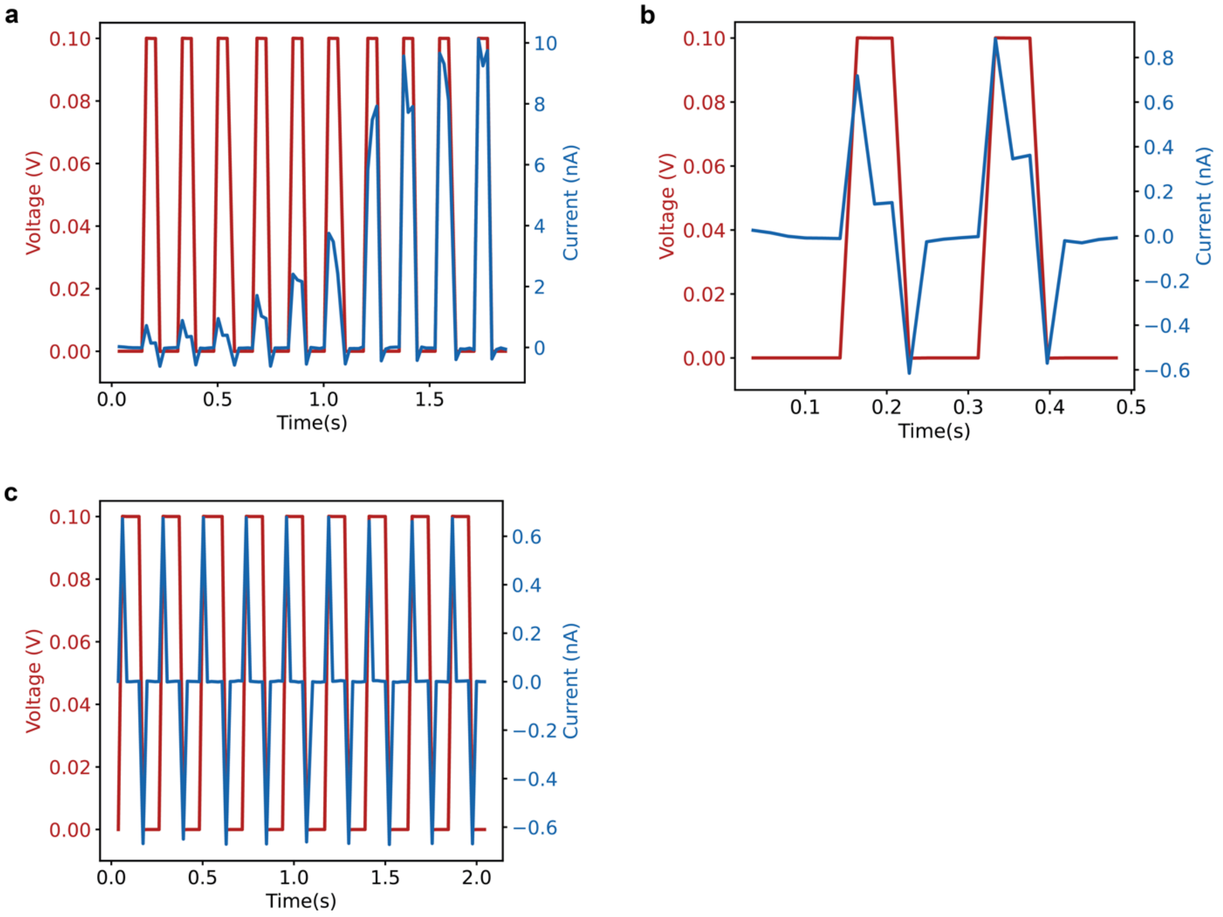

Figure S8. Uncorrected pulsed IV data. ***(a)*** *The full pulsed measurement, consisting of ten consecutive 0.1 V pulses of 55 ms in duration.* ***(b)*** *The first two pulses of* ***(a)****.* ***(c)*** *Pulsed measurement without contacting the sample.*

Supplementary Note 2. Considering crosstalk distance for high integration densities

At high integration density, crosstalk between neighboring devices might occur by formation of a conductive filament between top and bottom electrodes of two different, closely spaced devices. Taking into account that no switching occurs at 1 mV for a distance between the electrodes of 60 nm, it follows that there is no cross-talk of this type between devices so long as the electric field strength stays below approximately 17 kV/m. Assuming a uniform electric field between the electrodes so that $E = \frac{V}{d}$, where E is the electric field strength, V is the applied voltage and d is the distance between the electrodes, and assuming an operating voltage of 100 mV for each individual device, this implies that the distance between the devices would have to be 6 µm, or 100 times the distance between the electrodes of an individual device to prevent crosstalk. However, this consideration is relevant for vertical integration, yet in pracrtical devices the horizontal direction is more relevant for potential crosstalk.

To investigate horizontal crosstalk for densily integrated devices, we fabricated a lateral electrode device with 1 µm wide electrodes with a gap size of 90 nm (Figure S9a), comparable to the distance between the electrodes of the device in the main text. Interestingly, hysteresis was observed for the device, as shown in Figure S9b, but this hysteresis was similar to the hysteresis that is typically reported for halide perovskite solar cells.[1] No abrupt changes in the current over orders of magnitude was observed, even when applying potentials up to 5 V. The difference in the response between horizontally and vertically oriented devices might be explained by the preferential migration of ions at grain boundaries,[2] and the consequential preferential formation of conductive filaments at these grain boundaries.[3] Although more measurements are required to confirm the limit of the lateral distance between devices to avoid crosstalk, this measurement shows that crosstalk over a distance of 90 nm would not be significant compared to the resistance changes of the device presented in the main text. At 90 nm distance, very high device densities can be reached.

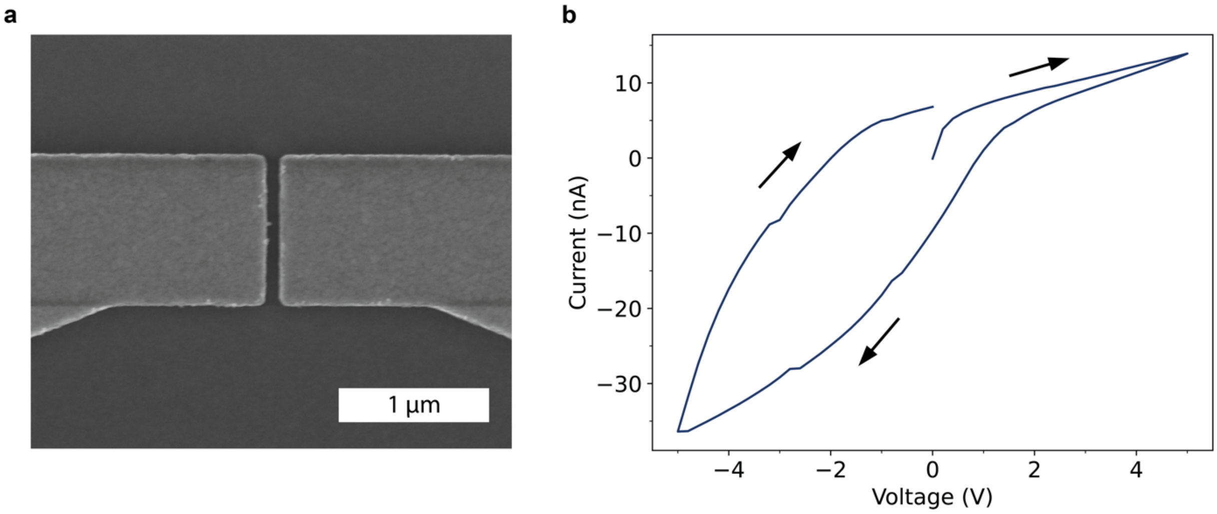

Figure S9. I-V sweep of a lateral device. ***(a)*** *SEM image of the lateral electrodes with a width of 1 µm and a 90 nm gap.* ***(b)*** *I-V sweep of the lateral device after spin coating of* $MAPbI_3$ *and PMMA. No abrupt changes of the resistance over orders of magnitude is measured for the lateral device, even when applying up to 5 V.*

Supplementary Note 3. Compilation of Figure 5

To indicate the performance of our halide perovskite artificial synapse, we compared the energy consumption, ON/OFF ratio, retention time, switching speed and multilevel operation from the references in Figure 5a and b on energy-efficient artificial synapses with those of our synapse. The data we used to score the different devices is summarized in Table S1. For Figure 5a and b in the main text, we plot the energy consumptions listed in Table S1 with respect to the reported device area and ON/OFF ratio. The exact device area of our device is difficult to define due to the arc-shaped electric field drop indicated in Figure 1, which is why we take the 2.5 x 2.5 μm^2 footprint of our device as an estimate. A linear decrease in energy consumption is expected as the device area is decreased further, due to the reduction in operating current. We indicate this linear decrease with the dashed line in Figure 5a. We note that taking a larger area for our device area to account for an electric field that extends further out from the

electrodes would shift the data point for our device further to the right in the figure, without changing the trend in decreasing energy consumption with device area.
In Figure 5c we compare our device performance to that of the devices reported in the references in Figure 5a and b. Retention times were not listed in the case of the phase change and ferroelectric devices. We therefore estimated the retention time based on other works on the same materials. $Ge_2Sb_2Te_5$-based phase change devices show similar retention times as the filamentary-type devices for the fully off and on states. However, there is typically considerable drift in the conductance of intermediate states at much shorter timescales of tens to hundreds of seconds.[4] Their retention time was therefore scored lower than that of the filamentary type devices. For the ferroelectric devices, previous retention times on the order of 10^4 seconds at room temperature were reported.[5]
To assess the scalability of the different devices, we considered that the filamentary and phase change devices have already been implemented with sizes below a square micron. The filamentary-type devices were scored lower because of the relatively high ON-state conductance, which limits the maximum amount of devices that can be operated in parallel in crossbar arrays. The ferroelectric devices were scored lower because of concerns about the depolarization of oxide perovskite ferroelectric layers below a critical thickness,[6] as well as the non-uniform polarization on the nanoscale for HfO_2-based devices.[7] We scored our device based on the high resistance, ease of further downscaling by further reducing the thickness of the electrodes and our initial data showing that the devices can be fabricated with a distance between devices below 100 nm without introducing significant crosstalk.

Table S1. Data on which the radar chart in Figure 5c in the main text is based.

Device type	Energy consumption	ON/OFF ratio	Retention time	Switching speed	Number of accessible states	Reference in the main text
Filamentary	100 fJ	10	≥10^5 s at 250 °C	10-100 ns	2	17
Filamentary	6 pJ	10^3	≥7200 s at 100 °C	10-30 ns	10	18
Phase change	1 pJ	10^3	not listed	250 ns	~100	19
Phase change	2 - 50 pJ	10^4	not listed	50 ns	~100	20
Ferroelectrics	~15 pJ	300	not listed	10-200 ns	~100	21
Ferroelectrics	1.8 pJ	5	not listed	100 ns	~10	22
**Our work**	**640 fJ**	**10^4**	**30 s**	**55 ms**	**~10**	**-**

References

(1) Chen, B.; Yang, M.; Priya, S.; Zhu, K. Origin of J–V Hysteresis in Perovskite Solar Cells. *J. Phys. Chem. Lett.* **2016**, *7* (5), 905–917. https://doi.org/10.1021/acs.jpclett.6b00215.

(2) Shao, Y.; Fang, Y.; Li, T.; Wang, Q.; Dong, Q.; Deng, Y.; Yuan, Y.; Wei, H.; Wang, M.; Gruverman, A.; Shield, J.; Huang, J. Grain Boundary Dominated Ion Migration in Polycrystalline Organic–Inorganic Halide Perovskite Films. *Energy Environ. Sci.* **2016**, *9* (5), 1752–1759. https://doi.org/10.1039/C6EE00413J.

(3) Lanza, M.; Bersuker, G.; Porti, M.; Miranda, E.; Nafría, M.; Aymerich, X. Resistive Switching in Hafnium Dioxide Layers: Local Phenomenon at Grain Boundaries. *Appl. Phys. Lett.* **2012**, *101* (19), 193502. https://doi.org/10.1063/1.4765342.

(4) Boniardi, M.; Ielmini, D.; Lavizzari, S.; Lacaita, A. L.; Redael, A.; Pirovano, A. Statistics of Resistance Drift Due to Structural Relaxation in Phase-Change Memory Arrays. *IEEE Trans. Electron Devices* **2010**, *57* (10), 2690–2696. https://doi.org/10.1109/TED.2010.2058771.

(5) Cheema, S. S.; Shanker, N.; Hsu, C.-H.; Datar, A.; Bae, J.; Kwon, D.; Salahuddin, S. One Nanometer HfO2-Based Ferroelectric Tunnel Junctions on Silicon. *Adv. Electron. Mater.* **2022**, *8* (6), 2100499. https://doi.org/10.1002/aelm.202100499.

(6) Junquera, J.; Ghosez, P. Critical Thickness for Ferroelectricity in Perovskite Ultrathin Films. *Nature* **2003**, *422* (6931), 506–509. https://doi.org/10.1038/nature01501.

(7) Chouprik, A.; Zakharchenko, S.; Spiridonov, M.; Zarubin, S.; Chernikova, A.; Kirtaev, R.; Buragohain, P.; Gruverman, A.; Zenkevich, A.; Negrov, D. Ferroelectricity in $Hf_{0.5}Zr_{0.5}O_2$ Thin Films: A Microscopic Study of the Polarization Switching Phenomenon and Field-Induced Phase Transformations. *ACS Appl. Mater. Interfaces* **2018**, *10* (10), 8818–8826. https://doi.org/10.1021/acsami.7b17482.
